# Supplementary material for: Climate Warming Does Not Override Eutrophication, but Facilitates Nutrient Release from Sediment and Motivates Eutrophic Process
Source: Microorganisms. 2023 Mar 31;11(4):910. doi: 10.3390/microorganisms11040910 (PMC10143447; doi:10.3390/microorganisms11040910)
Supplement: Supplementary file 1 [file microorganisms-11-00910-s001.zip › microorganisms-2241853-supplementary.pdf]

Supplementary information

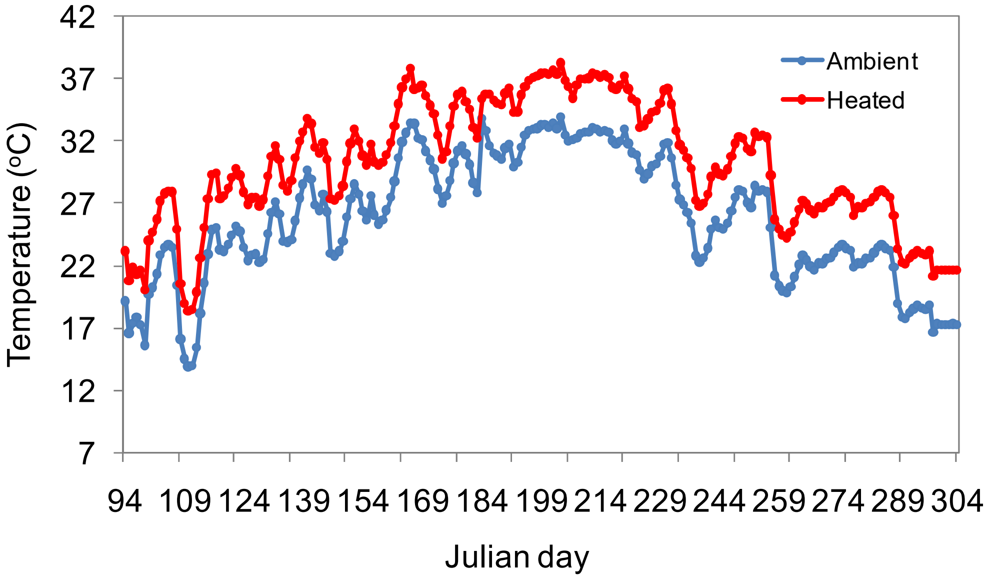

Figure S1. Changes in daily mean water temperature between April and October in 12 mesocosms warmed by 4.5°C above ambient and 12 unheated mesocosms.

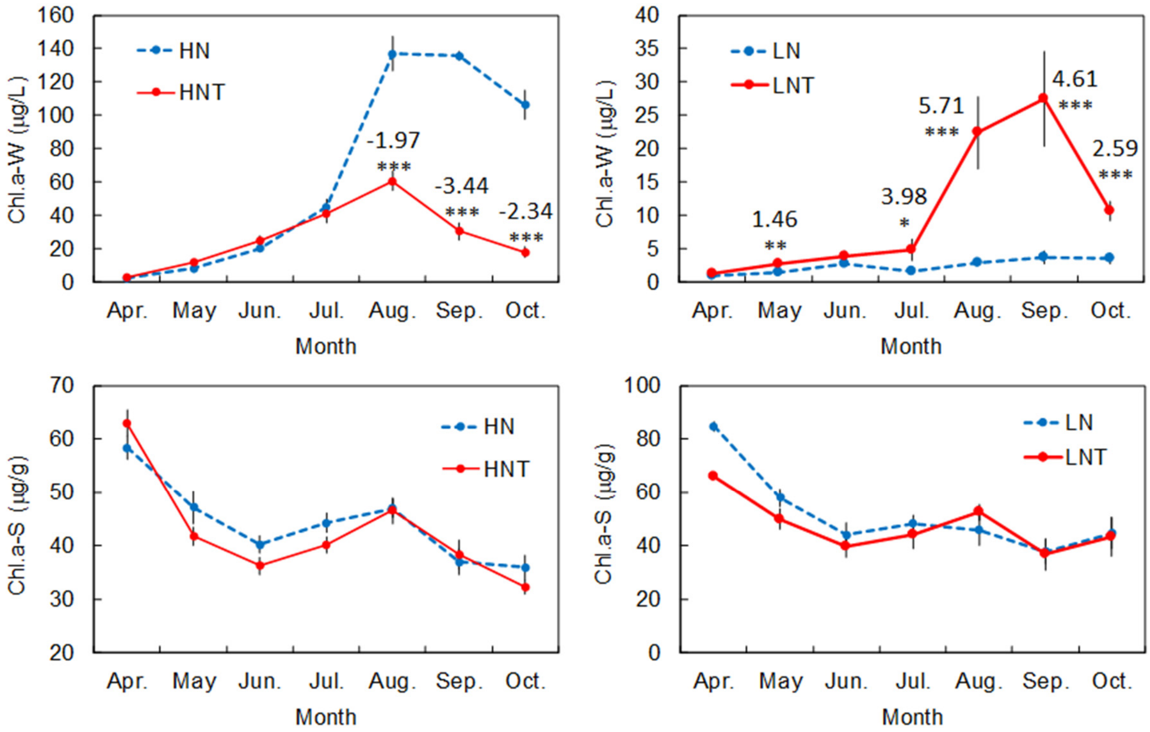

Figure S2. Changes in mean chl *a* of overlying water and sediment between April and October in 24 mesocosms subjected to different temperature (ambient + 4.5°C, ambient) under different nutrient levels (high, low). Shown are means subjected to different temperature (ambient + 4.5 different results (***: p<0.01, **: p<0.05, *: p<0.1 from ANOVAS for individual dates) for warming treatments. Numbers are effect sizes caused by warming on individual dates.

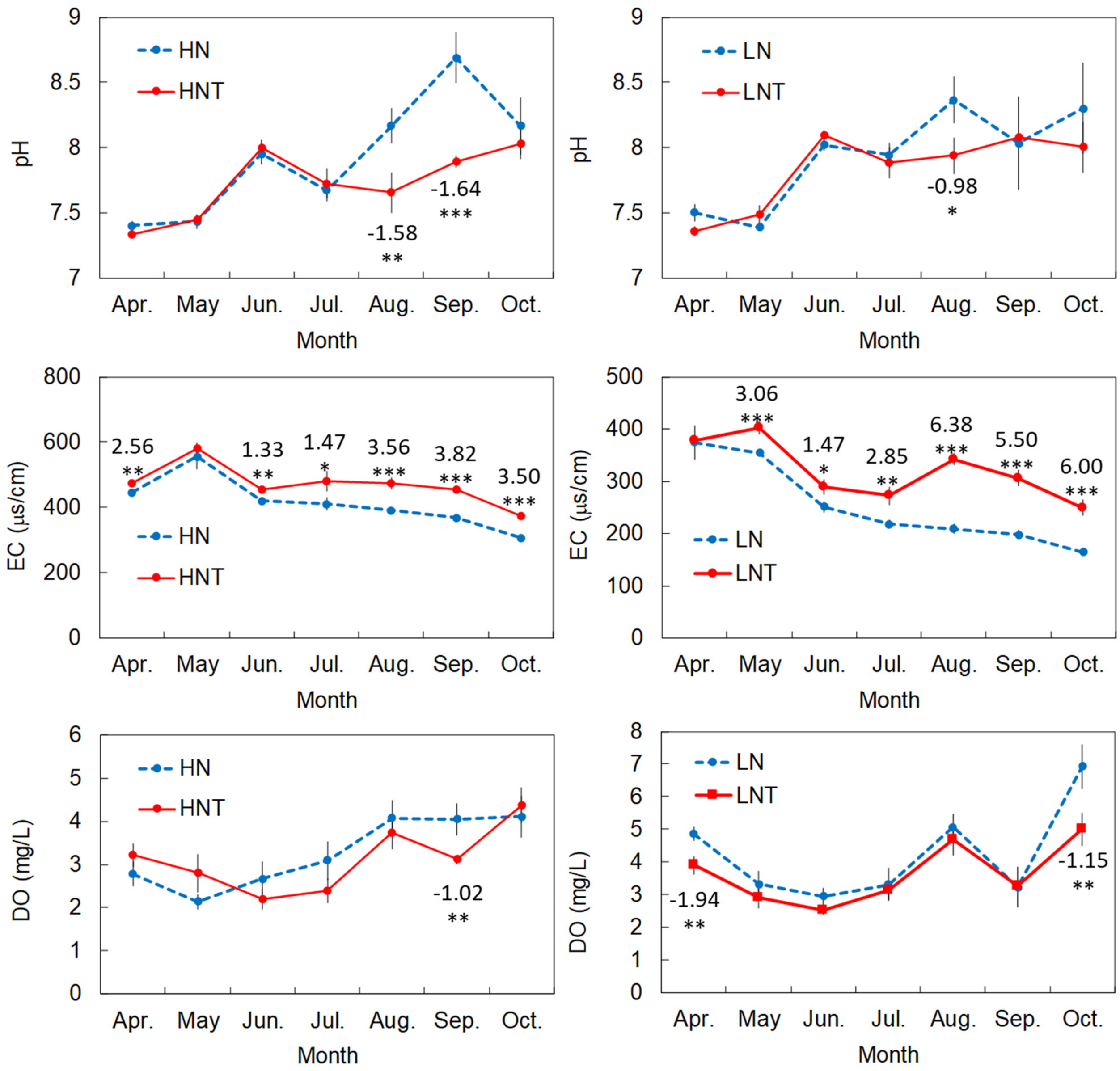

Figure S3. Changes in mean pH, EC and DO concentrations of overlying water between April and October in 24 mesocosms subjected to different temperature (ambient + 4.5°C, ambient) under different nutrient levels (high, low). Shown are means subjected to different temperature (ambient + 4.5 different results (***: *p*<0.01, **: *p*<0.05, *: *p*<0.1 from ANOVAS for individual dates) for warming treatments. Numbers are effect sizes caused by warming on individual dates.

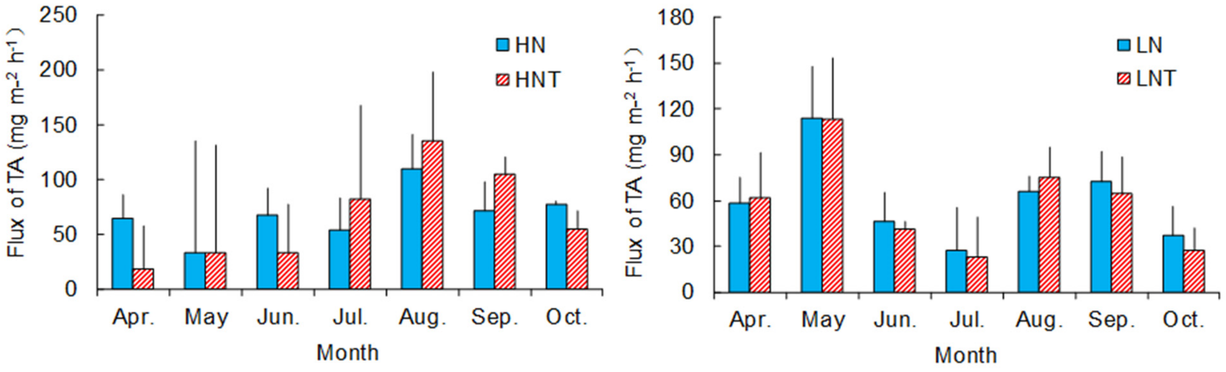

Figure S4. Sediment-water TA fluxes between April and October in 24 mesocosms subjected to different temperature (ambient + 4.5°C, ambient) under different nutrient levels (high, low). Shown are means + SE (n = 6).

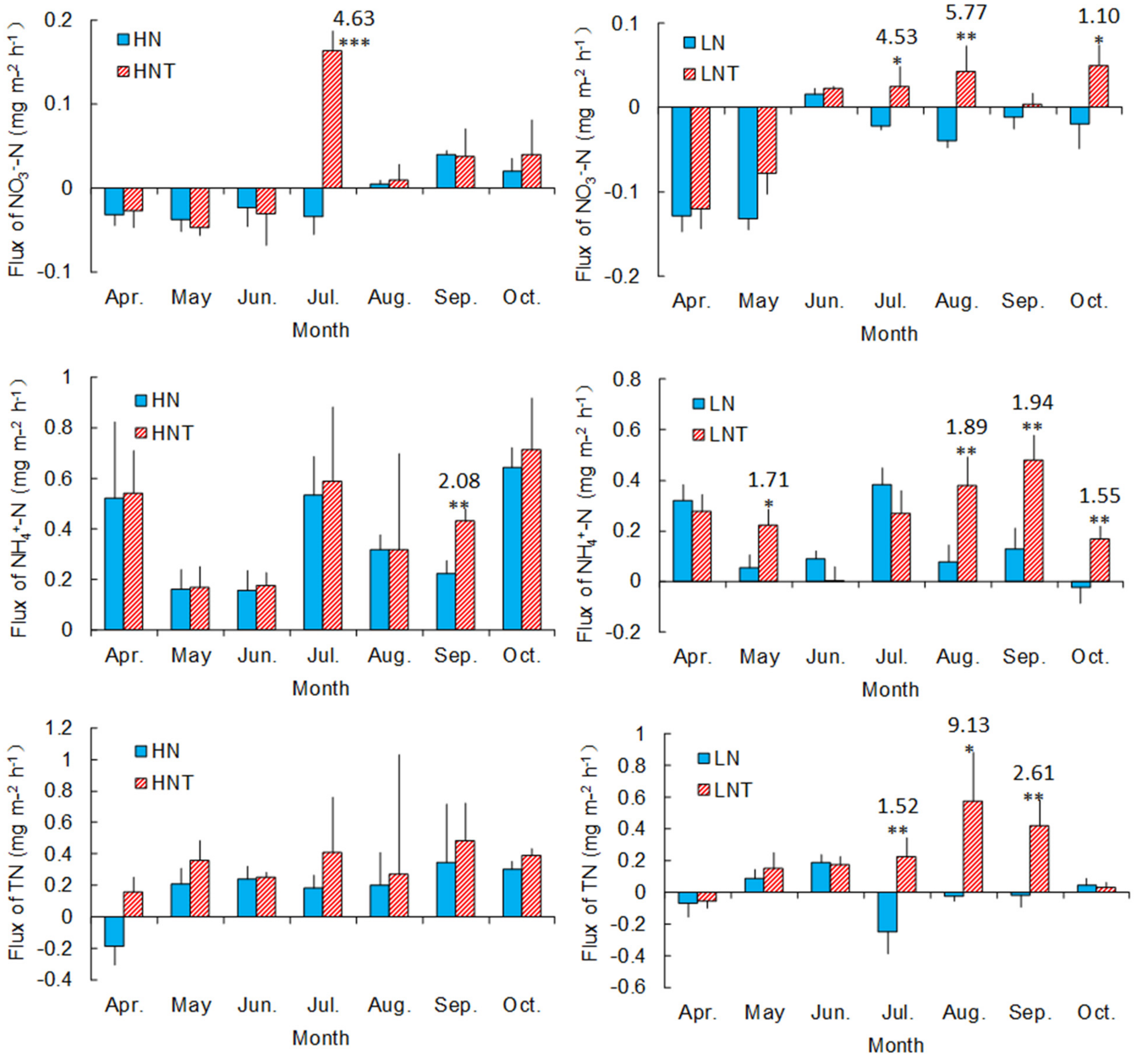

Figure S5. Sediment-water NH_4^+-N, NO_3^--N and TN fluxes between April and October in 24 mesocosms subjected to different temperature (ambient + 4.5°C, ambient) under different nutrient levels (high, low). Shown are means + SE (n = 6). Asterisks (*) indicate significantly different results (***: p<0.01, **: p<0.05, *: p<0.1 from ANOVAS for individual dates) for warming treatments. Numbers are effect sizes caused by warming on individual dates.

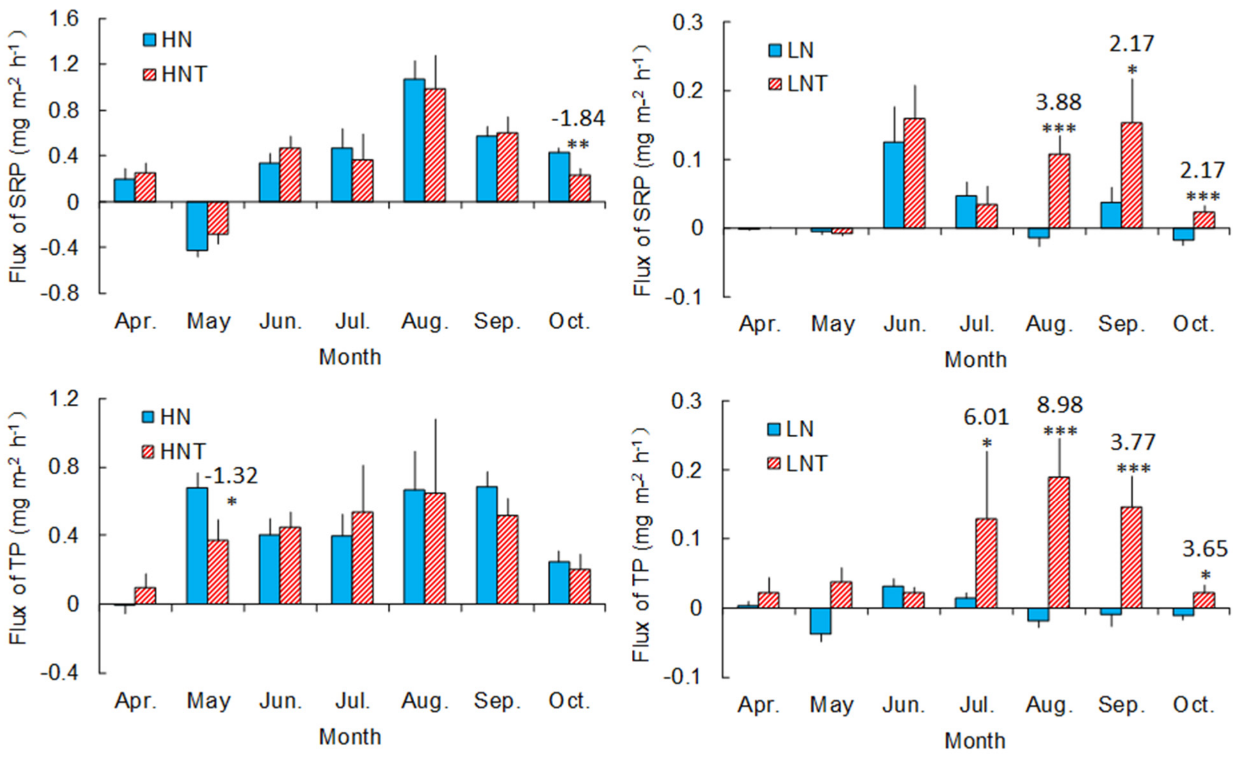

Figure S6. Sediment-water o-P and TP fluxes between April and October in 24 mesocosms subjected to different temperature (ambient + 4.5°C, ambient) under different nutrient levels (high, low). Shown are means + SE (n = 6). Asterisks (*) indicate significantly different results (***: p<0.01, **: p<0.05, *: p<0.1 from ANOVAS for individual dates) for warming treatments. Numbers are effect sizes caused by warming on individual dates.

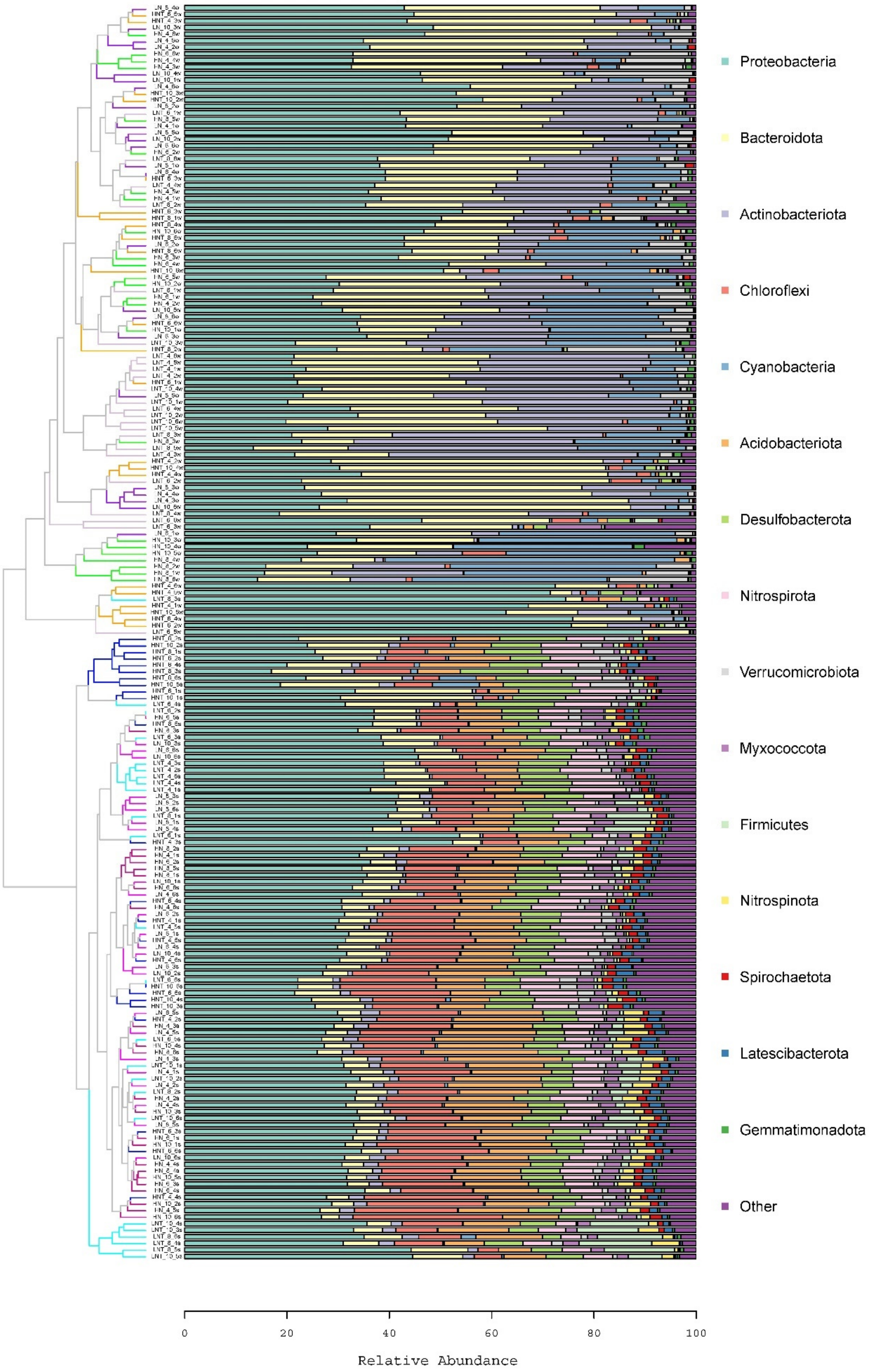

Figure S7. Relative abundance of top 15 phyla and other phyla along the sampling sites in water and sediment. The "w" represents water samples and "s" represents sediment samples. The phylum with an individual relative abundance of <1% or not in top 15 phylum of the analyzed samples is defined as the rare phylum.
